# Implementation of Mass Cytometry for Immunoprofiling of Patients with Solid Tumors

**DOI:** 10.1155/2019/6705949

**Published:** 2019-02-11

**Authors:** Ingrid Poláková, Ondřej Pelák, Daniel Thürner, Barbora Pokrývková, Ruth Tachezy, Tomáš Kalina, Michal Šmahel

**Affiliations:** ^1^Department of Genetics and Microbiology, Faculty of Science, Charles University, BIOCEV, Průmyslová 595, 25250 Vestec, Czech Republic; ^2^CLIP-Childhood Leukaemia Investigation Prague, Department of Paediatric Haematology and Oncology, Second Faculty of Medicine, Charles University and Motol University Hospital, V Úvalu 84, 15006 Prague 5, Czech Republic

## Abstract

Monitoring immune responses to solid cancers may be a better prognostic tool than conventional staging criteria, and it can also serve as an important criterion for the selection of individualized therapy. Multiparametric phenotyping by mass cytometry extended possibilities for immunoprofiling. However, careful optimization of each step of such method is necessary for obtaining reliable results. Also, with respect to procedure length and costs, sample preparation, staining, and storage should be optimized. Here, we designed a panel of 31 antibodies which allows for identification of several subpopulations of lymphoid and myeloid cells in a solid tumor and peripheral blood simultaneously. For sample preparation, disaggregation of tumor tissue with two different collagenases combined with DNase I was compared, and removal of dead or tumor cells by magnetic separation was evaluated. Two possible procedures of barcoding for single-tube staining of several samples were examined. While the palladium-based barcoding affected the stability of several antigens, the staining with two differently labeled CD45 antibodies was suitable for cells isolated from a patient's blood and tumor. The storage of samples in the intercalation solution for up to two weeks did not influence results of the analysis, which allowed the measurement of samples collected within this interval on the same day. This procedure optimized on samples from patients with head and neck squamous cell carcinoma enabled identification of various immune cells including rare subpopulations.

## 1. Introduction

Cancer generation and progression are critically affected by the host immune system. Therefore, the systemic and local detection and characterization of immune cells can be important for the evaluation of disease prognosis and prediction of the effect of available therapeutic options, including therapy harnessing the immune system.

Cancer immunotherapy was revitalized in recent years, and its clinical use is progressively increasing, especially after the US Food and Drug Administration (FDA) approval of the monoclonal antibodies ipilimumab, in 2011, and nivolumab and pembrolizumab, in 2014, targeting the immune checkpoints cytotoxic T lymphocyte-associated antigen 4 (CTLA-4; CD152) and programmed cell death protein 1 (PD-1; CD279), respectively. Besides these antibodies, other promising immunotherapeutic approaches against malignant diseases—adoptive transfer of modified T cells, cancer vaccines, and chimeric monoclonal antibodies called bispecific T cell engager (BiTE)—are now available [1, 2].

The development of cancer immunotherapy is associated with the detection of immune reactions, cells, and markers that enables the monitoring of the effect of therapy but is also important for prognosis and prediction of treatment success because only a minority of patients is responsive to immunotherapy. Moreover, immunomonitoring can also be beneficial for conventional cancer chemotherapy and radiotherapy as immune reactions can contribute to the effect of these treatment modalities [3].

Tumors are usually infiltrated by various types of immune cells that interact with tumor cells and influence tumor development. The assumption that the detection of these immune cells has a prognostic value led to the concept of “immunoscore” where immune cells are quantified in tumors by immunohistochemistry and their prognostic potential is evaluated. For early-stage colorectal cancer, the immunoscore seems to be a superior prognostic factor in comparison to tumor-node-metastasis (TNM) classification [4].

The immunoscore is mostly based on the detection of subpopulations of T lymphocytes, particularly cytotoxic CD8^+^ T cells [5], which are commonly supposed to be the major effector antitumor cells. However, at least in some tumors, other immune cells might play a crucial role in direct elimination of tumor cells [6, 7], and various immune cells are involved in complex regulation of immune reactions in the tumor microenvironment. Multiparametric phenotyping of immune cells from both tumors and peripheral blood can identify new markers for prognosis and monitoring the patient's immune status. Mass cytometry, capable of detecting over 40 parameters, is particularly suitable for such deep immunoprofiling [8].

In this study, we optimized sample preparation and staining for simultaneous analysis of immune cells in tumors and blood of patients with head and neck squamous cell carcinoma (HNSCC) by mass cytometry.

## 2. Materials and Methods

### 2.1. Sample Collection

Human blood samples of healthy volunteers were provided by the Institute of Hematology and Blood Transfusion in Prague and stored at room temperature (RT) after the collection and during the transport. Tumor tissue samples from tonsillar carcinoma were obtained from the Department of Otorhinolaryngology and Head and Neck Surgery of Motol University Hospital in Prague after the approval by the Institutional Review Board of the hospital and the obtainment of signature of the informed consent by patients. The tumor tissues were stored in RPMI medium (Sigma-Aldrich, St. Louis, MO) at 4°C during the transportation. Both types of samples were processed immediately upon the delivery.

### 2.2. Human Blood Cell Isolation

The Ficoll-Paque PLUS cell preparation protocol (GE Healthcare, Uppsala, Sweden) was used to obtain peripheral blood mononuclear cells (PBMCs) from noncoagulable (EDTA-treated) blood samples.

### 2.3. Tumor Cell Isolation

The tumor tissue was rinsed with phosphate-buffered saline (PBS), cut to pieces, and treated with 1 mg/ml collagenase D (Col D; Roche Diagnostics, Mannheim, Germany) or 1 mg/ml collagenase NB8 (Col NB8; SERVA, Heidelberg, Germany) and 100 *μ*g/ml DNase I (Roche Diagnostics) in RPMI medium. The gentleMACS Octo Dissociator with Heaters (Miltenyi Biotec, Bergisch Gladbach, Germany) and its h_tumor_01_01 and 37C_m_TDK_2 predefined programs were used to dissociate the tumor tissue at 37°C. Then, the samples were filtered through a 70 *μ*m strainer to get a single-cell suspension. Erythrocytes were removed by an ACK lysing buffer (0.15 M NH_4_Cl, 10 mM KHCO_3_, and 0.5 M EDTA (pH 7.2–7.4)).

### 2.4. Magnetic Separation of Cells

Cells labeled with antibodies conjugated to magnetic beads were separated by the autoMACS Pro Separator (Miltenyi Biotec). For disposal of dead cells from PBMCs or tumor cell suspensions, the Dead Cell Removal Kit (Miltenyi Biotec) was used. To concentrate tumor-infiltrating immune cells, epithelial tumor cells were removed after labeling with CD326 (EpCAM) Microbeads (Miltenyi Biotec). Before labeling, the Fc receptor of non-epithelial cells was blocked with FcR Blocking Reagent (Miltenyi Biotec). Positive and negative fractions obtained after separation of cells were fluorescently stained with monoclonal antibodies FITC anti-CD326 (clone HEA-125) and PE anti-CD45 (clone 5B1; both Miltenyi Biotec). The stained cells were measured on an LSRFortessa flow cytometer (BD Biosciences, San Diego, CA) and analyzed using the FlowJo v10.4.1 Software (BD Biosciences).

### 2.5. Mass Cytometry

To distinguish live and dead cells, blood and tumor samples were first stained with Cell-ID Cisplatin-^198^Pt (Fluidigm, South San Francisco, CA). Then, samples were barcoded with the Cell-ID 20-Plex Pd Barcoding Kit (Fluidigm). For this barcoding, samples underwent fixation (Fix I Buffer) and gentle permeabilization (Barcode Perm Buffer) to be labeled with the combination of three palladium isotopes. The labeling pattern for debarcoding is indicated in the user guide of the kit. Alternatively, samples were barcoded with two differently labeled anti-CD45 antibodies, CD45-156Gd and CD45-89Y (both clone HI30, Fluidigm), according to the Maxpar Cell Surface Staining protocol (Fluidigm). Cells were resuspended in 100 *μ*l of total staining volume of Maxpar Cell Staining Buffer with 1 *μ*l of antibody and incubated at RT for 30 minutes. Cell barcoding was followed by the staining of surface and nuclear antigens observing the procedure recommended by the Maxpar Nuclear Antigen Staining kit (Fluidigm). In brief, cells were washed by Maxpar Cell Staining Buffer after barcoding and incubated at RT for 30 minutes with the surface antibody cocktail in 100 *μ*l of total staining volume. The surface staining was followed by a washing step and incubation in 1 ml of Nuclear Antigen Staining Buffer for another 30 minutes. Then, two washing steps with Nuclear Antigen Staining Perm were performed, and the cells were stained with the intracellular antibody cocktail for 40 minutes. After another two washings with Maxpar Cell Staining Buffer, 1 ml of Cell-ID Intercalator-Ir solution was added to the cells. Antibodies (Table 1) obtained mostly from Fluidigm were preconjugated with metal isotopes. Some antibodies were purchased from other companies and required conjugation with isotope tags applying the Maxpar Antibody Labeling Kit (Fluidigm). Stained and fixed cells were stored at 4°C until ready to run on a CyTOF2 mass cytometer (Fluidigm). The data were exported as flow cytometry file (.fcs) format.

## 3. Results

### 3.1. Designing a Panel of 31 Antibodies for Immunomonitoring of Cancer Patients

We designed a panel of antibodies for multiparametric phenotyping of blood and tumor samples of patients with malignant diseases. Using the Maxpar Panel Designer (Fluidigm), we created a panel of 31 antibodies suitable for mass cytometry (Table 1). In this panel, we focused on various subpopulations of T lymphocytes (naïve, activated, and memory cells and effector and regulatory cells) and myeloid cells (monocytes, macrophages, and dendritic cells). Additionally, B cells and NK cells can also be identified.

### 3.2. Collagenase D Was Suitable for Enzymatic Dissociation of the Tumor Tissue

**Figure 1:**
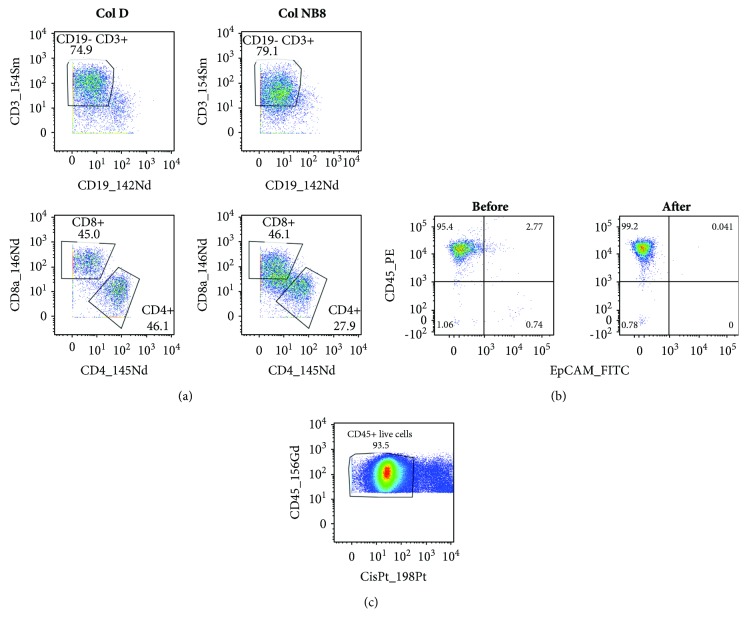
Sample preparation. (a) Tumor tissue was halved and treated with either Col D or Col NB8 together with DNase I using a gentleMACS Octo Dissociator and its predefined programs. After lysis of erythrocytes, the single-cell suspensions were stained with the designed panel of antibodies. The same gates were applied to cell populations originated from the tissues treated with Col D or Col NB8. (b) Samples of cells prepared from tumors were labeled with fluorescent anti-CD45 and anti-EpCAM antibodies before and after magnetic removal of epithelial cells. (c) Cells isolated from the tumor sample were stained with cisplatin for viability detection. The experiments were repeated with similar results.

To optimize the staining of cells for mass cytometry, we first optimized sample preparation. As we plan to apply the established procedure for the analysis of tumors from patients with HNSCC, we used samples from tonsillar carcinomas in the optimization part of the study. For isolation of single cells from these tumors, we combined mechanical and enzymatic disaggregation techniques. Collagenase and DNase I are enzymes commonly used for the dissociation of tumor tissues, and their applicability for mass cytometry has recently been demonstrated [9]. Based on our prior experiences with the usage of Col NB8 (SERVA) for preparation of tumor-infiltrating cells from mouse tumors [10] and Col D (Roche) for HNSCC [11], we compared these two types of collagenases. Tumor samples were divided into two parts of the same weight and processed in the gentleMACS Octo Dissociator using predefined programs with the enzymatic treatment at 37°C for 40 min. Regardless of the collagenase used, the total numbers of cells and their viability were repeatedly comparable. Isolated cells were stained with the designed panel of antibodies and evaluated by mass cytometry. We found that the treatment with Col D better preserved the detected antigens as documented in Figure 1(a) by gating of CD3^+^, CD4^+^, and CD8^+^ cells. As the determination of these crucial cell populations was nearly impossible after the treatment of the tumor tissue with Col NB8, we decided to use Col D for further analysis.

### 3.3. Removal of Dead Cells and Epithelial Tumor Cells from the Dissociated Tumor Tissue Was Not Usually Necessary

In comparison with flow cytometry, measurement of samples by a mass cytometer is more time-consuming (because of a low acquisition rate) and more expensive (because of argon consumption). Therefore, a high proportion of appropriate cells in measured samples are desirable. Single-cell suspensions prepared by dissociation of tumors contain various numbers of tumor-infiltrating immune cells and epithelial tumor cells that can further differ in viability. To concentrate live tumor-infiltrating immune cells, we considered the removal of dead cells and/or epithelial tumor cells by magnetic separation on autoMACS after labeling of annexin V or EpCAM, respectively. The labeled cells were efficiently removed, but about a half of all cells were lost during this procedure. With respect to a low portion of EpCAM^+^ cells (usually less than 15%; Figure 1(b)) and dead cells (mostly about 20–35%) in single-cell suspensions prepared from tumors and high loss of cells in magnetic separations, we omitted the concentration of immune cells in the following experiments. However, to distinguish live and dead cells in the sample, the cells were labeled with cisplatin, containing the ^198^Pt isotope, before surface and intracellular staining (Figure 1(c)).

### 3.4. Pd-Based Barcoding Affected Stability of Several Antigens

**Figure 2:**
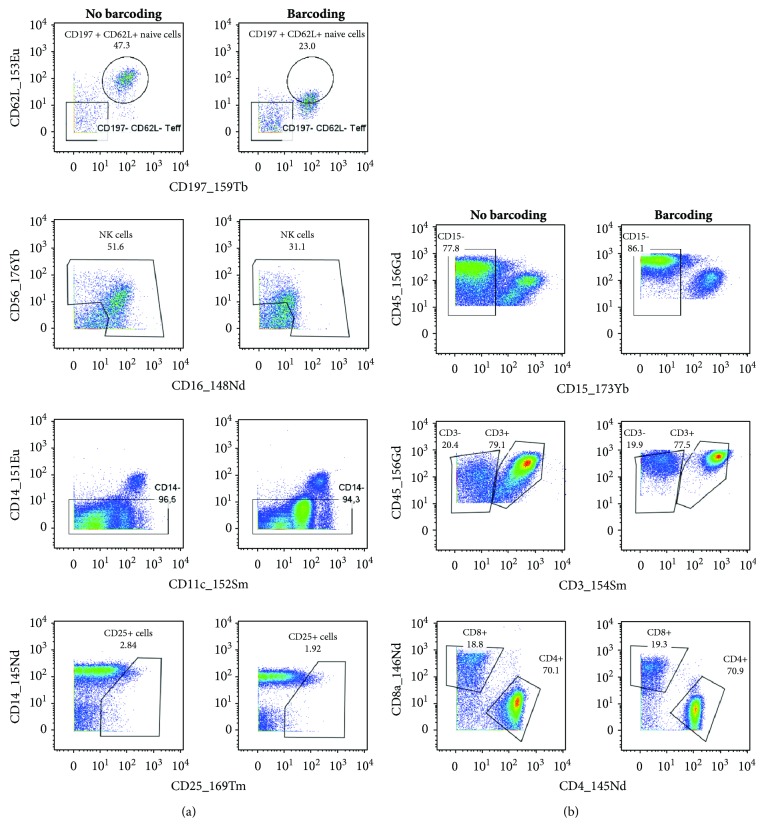
Stability of several antigens was affected by Pd-based barcoding. Ficoll-isolated PBMCs from a healthy donor were stained for live and dead cells and halved. One part of the cells was barcoded with the Cell-ID 20-Plex Pd Barcoding Kit, and the rest of the cells were used as a control without barcoding. Subsequently, the cells were stained with the designed panel of antibodies. The labeled cells were stored in the intercalator solution until measurement on CyTOF. After measurement, the barcoded cells were debarcoded according to the labeling pattern of the barcoding kit. (a) Markers with reduced intensity and (b) cell populations become more evident after Pd barcoding. The same gates (adjusted for the cells without barcoding) were applied to measured cells.

To decrease high costs of cell labeling and sample measurement, we tested a single-tube staining of cells obtained from multiple samples and analyzed tumor and blood cells from several patients together. To distinguish cells isolated from blood and tumors and from different patients, a commercial barcoding kit based on palladium (Pd) isotopes was first tested. This kit is able to barcode, subsequently stain, and measure up to 20 samples as a one multiplexed sample. Samples were labeled with a combination of three Pd isotopes after fixation and gentle permeabilization steps. The barcoded samples were then combined in a single tube and stained with the designed panel of antibodies. After analysis of staining intensity, we observed a considerable population shift for some markers when comparing the barcoded samples with samples without barcoding (Figure 2). CD62L, CD16, CD56, CD11c, and CD25 were the markers with the most prominent reduction of positivity after barcoding, which disabled proper identification of cell populations (Figure 2(a)). On the contrary, the boundary between some populations (CD15^+^, CD3^+^, CD4^+^, and CD8^+^; Figure 2(b)) became more evident. As our main intent is to preserve the integrity of all cellular markers, the Pd-based barcoding technique is not applicable to our panel of antibodies.

### 3.5. CD45-Based Barcoding Did Not Affect Detection of Cell Markers

**Figure 3:**
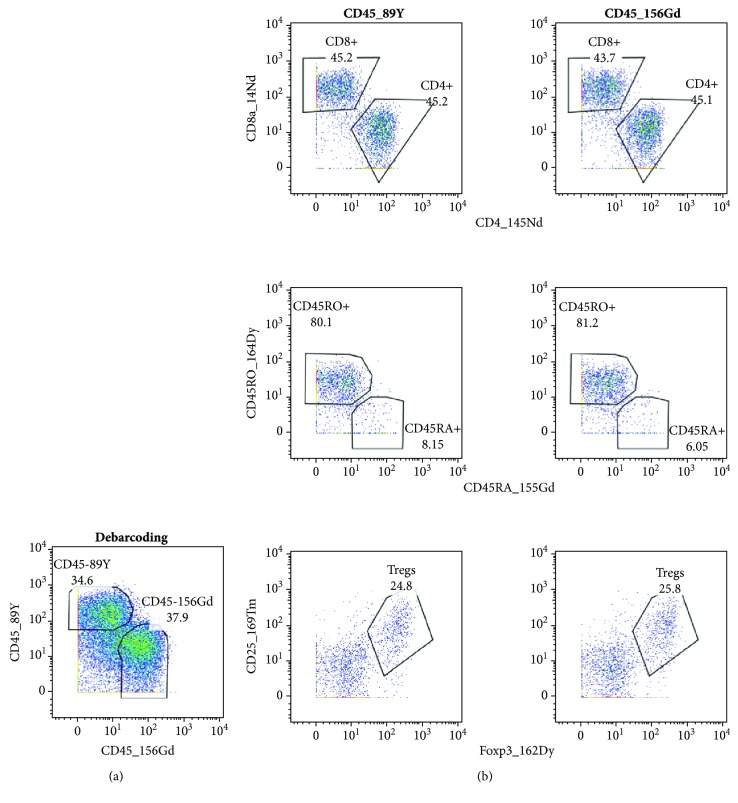
CD45-based barcoding. A single-cell suspension was prepared from tonsillar carcinoma, stained for live and dead cells, and halved for barcoding. After barcoding with CD45-89Y and CD45-156Gd antibodies, the samples were combined and stained with the rest of the antibodies. (a) Debarcoding and (b) dot plots with a selection of representative antigens. The same gates were applied to measured cells.

Antibody against CD45 molecules labeled with different isotopes is another possibility of sample barcoding [12]. To distinguish between cells from blood and tumors, we tested two metal-labeled antibodies (CD45-156Gd and CD45-89Y). For comparison of their staining efficacy and their effect on the detection of other cell markers, we halved the cells from a tumor sample and barcoded them with the CD45 antibodies. Subsequently, the barcoded cells were combined and stained together with the remaining antibodies from the CyTOF panel.  Figure 3 demonstrates that antibodies against CD45 identified practically identical cell populations, and parallel samples labeled with these antibodies did not differ in cell subpopulations detected by other antibodies. In view of these results, we decided to apply CD45-based barcoding to our samples.

### 3.6. Sample Storage Conditions

The sample preparation, barcoding, staining, and measurement are time-consuming procedures that can be further complicated by the fact that sample collection, processing, and analysis are carried out at different sites. This long procedure might be interrupted in a few points. One possibility is to stop the procedure after sample preparation. Simultaneously with testing the Pd-based barcoding, we attempted to store the prepared samples in Maxpar Fix I Buffer that is a component of the barcoding kit and is used for treatment of cells prior to the barcoding itself. After storing the samples in Fix I Buffer at 4°C overnight, changes obtained after Pd-based barcoding were intensified, and staining of several other antigens was affected (data not shown). Therefore, the Fix I Buffer turned out to be unsuitable for sample storage.

**Figure 4:**
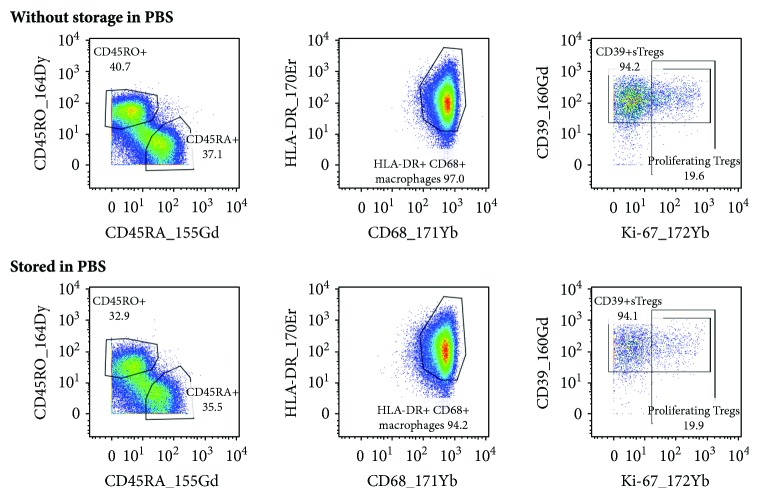
Sample storage in PBS with 1% BSA. Tumor cells and PBMCs were isolated from a patient with HNSCC and stained for viability. After CD45-based barcoding, samples were halved. One part of the cells was stained with the panel of antibodies immediately, and the other part was stored in PBS with 1% BSA at 4°C overnight and labeled the next day. Dot plots display some representative markers of the blood sample. Gates were adjusted for cell samples stained immediately after isolation.

As another option, we examined storing the CD45-barcoded samples in PBS with 1% BSA at 4°C overnight. We found that this way of storage did not have any impact on the antigen stability and population gating (Figure 4).

**Figure 5:**
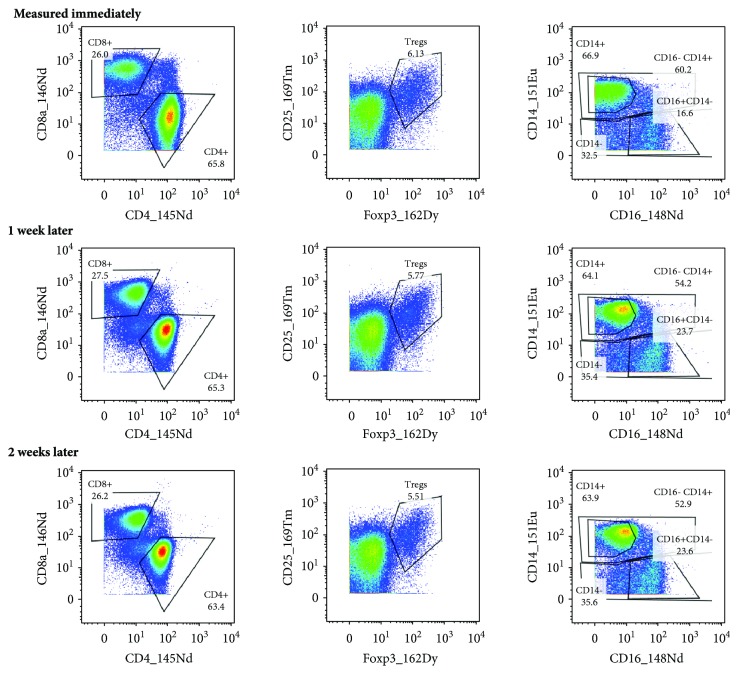
Storage of labeled samples in the intercalator solution. PBMCs were isolated from a blood sample of a patient with HNSCC, stained for live and dead cells, and divided for further labeling. The stained cells were measured the next day or stored in the intercalator solution for one or two weeks. The same gates (adjusted for the sample measured immediately) were applied to measured cells.

Collecting several samples labeled on different days to measure them at a time can also reduce the measurement costs and may be useful when a limited number of samples are stained per day. Sample staining terminates with resuspending the cells in an intercalator solution, which is intended to store the samples at 4°C until the measurement. We compared samples stored in the intercalator solution for one or two weeks with a sample measured immediately (on the next day) after labeling. As we did not observe any differences among these samples (Figure 5), we considered appropriate to store the labeled samples in the intercalator solution for at least two weeks.

## 4. Discussion

Mass cytometry with its capability to detect a large number of parameters is a convenient method for accurate immunoprofiling of cancer patients. Exposing the phenotype of the immune cells may contribute to disease prognosis and treatment selection. Therefore, we designed a panel of antibodies to detect a wide range of lymphoid and myeloid subpopulations in blood and tumor samples and optimized the procedure of sample preparation and labelling for simultaneous analysis of peripheral blood cells and immune cells isolated from a solid tumor of the same patient.

Whereas a standard Ficoll gradient centrifugation protocol exists for PBMC separation, the storage conditions of blood samples until processing had to be considered. The drawn blood should be stored at RT and processed within 8 hours to avoid granulocyte contamination during PBMC isolation and to preserve T cells [13]. Moreover, it has been proven that the prolonged sample storage at RT before testing may cause a contact-dependent exchange of the CD3 antigen between T and B cells [14]. Fortunately, these phenomena were not observed in our experiments due to the immediate sample processing after delivery.

The tumor tissue, transported in RPMI medium on ice, was likewise promptly processed. The tissue dissociation (mechanically and/or enzymatically), leading to a single-cell suspension, is an essential step in immunoprofiling of solid tumors. As mass cytometry was developed recently, only few articles deal with tumor dissociation methods. To preserve the viability, integrity, and functionality of cells infiltrating a tumor, it is necessary to choose a suitable enzyme and determine the timing of tumor processing. Leelatian and his group compared several collagenases and DNases and their combinations [9] and proposed a protocol for human tumor dissociation [15]. Based on our previous experience with mouse and human tumor tissues, we compared the combination of DNase I with Col D or Col NB8. Although the viability of the cells isolated from tumors was similarly preserved in both combinations and the yield was comparable, it was not possible to define the basic cell populations after treatment with Col NB8. Consequently, the combination of DNase I and Col D was applied in further experiments.

Besides its many advantages, mass cytometry has two crucial limitations. The experiments carried out and the measurements may be costly and time-consuming. Therefore, it is essential to measure primarily the cells of our interest. To meet this requirement, we tried to remove dead and epithelial cells from the tumor samples prior to labeling. However, magnetic depletion of dead cells or EpCAM^+^ cells led to significant loss of desired population of viable immune cells. Additionally, flow cytometric analyses of several samples showed that the viability of isolated cells was high (usually >60%) and the portion of epithelial cells in samples from tonsillar carcinomas was mostly negligible. Therefore, we decided to remove only dead cells whenever their proportion was higher than 50% of all cells and the number of isolated cells was sufficient (at least 6 × 10^6^). Cisplatin staining prior to surface labeling was chosen to gate live and dead cells.

The staining and measurement costs could be further pushed down by a single-tube analysis of tumor and blood cells from several patients. Mass-tag cellular barcoding (MCB) techniques have been developed for experiment multiplexing. These techniques are based on lanthanide isotopes loaded on the compound maleimido-mono-amide-DOTA (mDOTA) [16], isothiocyanobenzyl-EDTA-loaded palladium isotopes [17], or osmium and ruthenium isotopes [18]. In our experiments, we tested the commercially available Pd-based barcoding kit, which is able to barcode up to 20 samples, but this kit was not usable in combination with our antibody panel as the staining procedure affected the stability of several antigens of our interest. Our following measurements revealed (data not shown) that at least 1.5 × 10^6^ (ideally 2.5 × 10^6^) cells isolated from both blood and tumor need to be stained to collect also the rare populations of cells. Moreover, as it was concluded that more than 4 × 10^6^ cells cannot be stained in a single tube, our only option was a combination of blood and tumor cells from only one patient into a single tube. Since it was sufficient to apply two barcodes for this purpose, we tested a multiplexing method directed against the cell surface-expressed CD45 [12, 19] that does not require fixation and partial permeabilization unlike the Pd-based barcoding. Staining of the blood and tumor samples originated from one patient with two differently labeled anti-CD45 antibodies was satisfying—none of the antigens was affected by the barcoding.

As the time from sample collection to its processing, staining, and measurement may vary, we searched for pause points in this long procedure. Prolonged incubation in the Fix I Buffer that is applied during the Pd-based barcoding could be a suitable way of interruption. However, overnight incubation of samples in this transient fixation buffer influenced the stability of several antigens. This result suggests that the effect of the Fix I Buffer is the reason why Pd barcoding is not applicable to our antibody panel. Another option was to store the isolated, viability-stained, and barcoded cells in PBS with 1% BSA at 4°C overnight before staining with the panel of antibodies. This delay in sample processing did not affect staining results when compared to the samples labeled immediately after barcoding. Accordingly, it was possible to interrupt the sample staining, under time pressure, via storing the barcoded cells in PBS with BSA.

The very last step of sample staining is resuspension of the labeled cells in the intercalation solution, which labels the nucleated cells with iridium isotopes. This procedure requires fixation and permeabilization of the cells. Using the Maxpar Nuclear Antigen Staining kit (Fluidigm), the intercalator is diluted in Fix & Perm Buffer that contains paraformaldehyde. The manufacturer's recommendation is to store the cells in the intercalator solution at 4°C until measurement on a mass cytometer, but no longer than 48 hours. However, Zunder and his group showed that it was possible to keep the samples in an in-house-prepared intercalation solution at 4°C for up to one month [17]. Therefore, we prolonged the sample storage in the intercalation solution to one and two weeks and compared the outcome with the sample measured the next day after labeling. As no differences were found among the compared samples, we concluded that the storage of the labeled cells in this solution is acceptable for at least two weeks.

## 5. Conclusions

A panel of 31 antibodies was designed for mass cytometry immunoprofiling of blood and tumor samples from patients with HNSCC. The method of tumor tissue dissociation was optimized by the combination of DNase I with the proper collagenase, and suitable barcoding of cells isolated from blood and tumor samples originated from one patient was incorporated in cell labeling. Next, a possible pause point in the long sample processing was found, and the storage of labeled samples until measurement on a mass cytometer was solved. Consequently, all surface and intracellular antigens of our interest were preserved and procedure costs were minimized.

## Figures and Tables

**Table 1:** Panel of antibodies for mass cytometry.

#	Antigen	Isotope	Clone	Staining	Company
1	CD1c (BDCA-1)^∗^	166Er	AD5-8E7	Surface	Miltenyi Biotec
2	CD3	154Sm	UCHT1	Surface	Fluidigm
3	CD4	145Nd	RPA-T4	Surface	Fluidigm
4	CD8a	146Nd	RPA-T8	Surface	Fluidigm
5	CD11b (Mac-1)	209Bi	ICRF44	Surface	Fluidigm
6	CD11c^∗^	152Sm	Bu15	Surface	BioLegend
7	CD14	151Eu	M5E2	Surface	Fluidigm
8	CD15^∗^	173Yb	MEM-158	Surface	Exbio
9	CD16	148Nd	3G8	Surface	Fluidigm
10	CD19	142Nd	HIB19	Surface	Fluidigm
11	CD25	169Tm	2A3	Surface	Fluidigm
12	CD39	160Gd	A1	Surface	Fluidigm
13	CD45	89Y or 156Gd	HI30	Surface	Fluidigm
14	CD45RA	155Gd	HI100	Surface	Fluidigm
15	CD45RO	164Dy	UCHL1	Surface	Fluidigm
16	CD56 (NCAM)	176Yb	N901	Surface	Fluidigm
17	CD62L (L-selectin)	153Eu	DREG-56	Surface	Fluidigm
18	CD68	171Yb	Y1/82A	Intracellular	Fluidigm
19	CD69	144Nd	FN50	Surface	Fluidigm
20	CD127 (IL-7R*α*)	149Sm	A019D5	Surface	Fluidigm
21	CD141 (BDCA-3)^∗^	165Ho	AD5-14H12	Surface	Miltenyi Biotec
22	CD152 (CTLA-4)	161Dy	14D3	Intracellular	Fluidigm
23	CD197 (CCR7)	159Tb	G043H7	Surface	Fluidigm
24	CD275 (ICOS-L)^∗^	163Dy	2D3/B7-H2	Surface	BD Biosciences
25	CD278 (ICOS)	143Nd	DX29	Surface	Fluidigm
26	CD279 (PD-1)	175Lu	EH12.2H7	Surface	Fluidigm
27	CD303 (BDCA-2)	147Sm	201A	Surface	Fluidigm
28	CD366 (Tim-3)	150Nd	F38-2E2	Surface	BioLegend
29	Foxp3	162Dy	259D/C7	Intracellular	Fluidigm
30	HLA-DR	170Er	L243	Surface	Fluidigm
31	Ki-67	172Yb	B56	Intracellular	Fluidigm

^∗^In-house conjugation with isotope tags applying the Maxpar Antibody Labeling Kit (Fluidigm).

## Data Availability

The data used to support the findings of this study are available from the corresponding author upon request.
